# The Urgency of Stress Reduction Amongst Medical Students: Is Therapeutic Touch the Answer? A Literature Review on Therapeutic Touch in Students and Healthcare Personnel

**DOI:** 10.1007/s40670-025-02463-z

**Published:** 2025-07-24

**Authors:** Gabrielle K. Sharbin, Keith Metzger

**Affiliations:** Department of Medical Sciences, Hackensack Meridian School of Medicine, Nutley, NJ USA

**Keywords:** Reiki, Therapeutic touch, Medical students, Stress, Medical education

## Abstract

Medical students face disproportionately high levels of stress, which can affect both their well-being and patient care. While mindfulness has shown mixed results, therapeutic touch and Reiki have demonstrated potential in reducing anxiety and depression. This review searched PubMed, Scopus, PsychInfo, Cochrane Library, and gray literature for studies on therapeutic touch in students and healthcare workers. Of 363 articles, 8 met inclusion criteria. Five of seven eligible studies (71.4%) reported statistically significant stress reduction. These findings suggest that therapeutic touch may benefit medical students, warranting further research into its potential as a stress reduction intervention.

## Background

It is well known that a salient problem facing medical students is the stress associated with rigorous medical training. This stress can lead to burnout, academic failure, and psychological implications such as depression and anxiety. Several studies have documented high levels of stress, anxiety, and depression amongst medical students [1, 2]. The global prevalence of anxiety in medical students is 33.8% [1], a rate substantially higher than that of the general population. According to the World Health Organization, 4% of the global population struggles with an anxiety disorder [3]. Similarly, medical students experience depression at disproportionately high rates, with 28% affected globally [2], compared to 5% of the general population [4].

These findings indicate a pressing need for effective stress reduction strategies targeted at medical students. Moreover, the growing burden of stress amongst this population is not only of individual concern, but it intersects with broader systemic challenges such as our nation’s upcoming physician shortage. It is well known that the US population is aging, and paralleling the expected rise in average age is projected decline in the number of practicing physicians. According to a report released by the Association of American Medical Colleges (AAMC), “*The Complexities of Physician Supply and Demand: Projections From 2019 to 2034*,” the USA faces a predicted shortage of 37,800–124,000 physicians within the next 12 years [5].

The catalyst for the fall in medical professionals has been speculated by many, and the reasons underlying this fragility in our medical system are stratified and complex. The projected shortage is multifactorial, driven by population growth, an aging population, physician retirement trends, rising rates of chronic disease, and limitations in medical education capacity [5]. The US population is expected to grow by 8.4% by 2036 [5], and the number of individuals aged 65 and older is expected to increase by 34.1% [5]. Accompanying the rise in the elderly population, undoubtedly comes a rise in patients requiring complex medical care for chronic diseases, further escalating demand for physicians. However, as the demand grows, the physician workforce is diminishing. Currently, 20% of practicing physicians are over 65 years old [5] and 22% are between the ages of 55 and 64. As these physicians retire, the supply of active practitioners will decrease, aggravating the shortage.

Despite the establishment of over 30 new MD-granting institutions, and a 35% rise in medical school enrollment since 2002, the number of residency positions has not kept pace with this growth. This is largely due to federal caps on Medicare support for graduate medical education (GME) that have been in place since 1997 [6]. This barrier limits the number of new physicians who can enter the workforce. Certain underserved geographic regions and demographic groups experience this shortage more acutely and are expected to confront even more severe gaps in care [5]. Additionally, disparities exist amongst specialties. While shortages are projected in primary care, surgical specialties, and medical specialties such as critical care/pulmonology and endocrinology, surpluses are expected in emergency medicine and physical medicine and rehabilitation [5].

The rigors associated with medical training, which contribute to burnout and attrition, are attached to this projected shortage as well [7]. Physicians experiencing burnout work fewer hours and are more likely to leave clinical medicine [7]. This underscores the urgency of addressing stress early on in the course of training future physicians. Our obligation to imminently address stress amongst medical students is not just to prevent a future physician shortage. Stress amongst medical students also directly impacts patient care. The quality of healthcare delivery is influenced in part by the abilities and knowledge of providers, and the pathway to becoming compassionate, giving, intellectually capable physician begins in medical school. This learning is incumbent upon the willingness and ability of medical students to engage fully in pre-clinical and clinical training. However, meaningful student learning can only occur when the stress of exams, school, and life does not interfere [8]. Additionally, provider stress has been shown to negatively impact patient care, contributing to medical errors, reduced quality of care, increased costs, and worse outcomes [7].

Mindfulness-based interventions have been utilized to help mitigate stress amongst medical students. However, results have been mixed, according to a 2017 systematic review [9]. This highlights the need to explore other potential interventions. Reiki provides a potential solution. Reiki, meaning “universal life energy,” is an ancient Japanese healing method that aims to manipulate energy flow in the body [10]. It involves a practitioner placing their hands lightly on or just above the body in a series of positions, with the intention of channeling healing energy to promote balance and well-being [11]. The practitioner moves through hand placements, often starting at the head and progressing down the body. Some forms involve direct touch, while others use hands-off techniques. The practice is believed to activate the body’s relaxation response, decrease stress, and support emotional and physical healing [11]. Reiki is acknowledged by the National Center for Complementary and Alternative Medicine [10] and is considered a non-invasive, low-risk, complementary therapy.

Energy therapies are defined by the National Center for Complementary and Alternative Medicine as methods of healing involving the conduction of healing energy through the hands of a practitioner into the recipient’s body, with the purpose of restoring homeostasis and promoting health [10]. Reiki practitioners believe that there is a flow of energy between the practitioner and the recipient, and that Reiki allows the body’s own natural healing ability to function more effectively [12]. Other examples of energy therapies include healing touch, Qigong, and homeopathy.

Western medical schools already teach the importance of physical touch in healing, with the physical exam itself seen as therapeutic. Reiki shares this sentiment, as it is typically administered through the “laying on of hands,” either through direct or indirect physical contact. Although Reiki has not yet been studied in medical students, it has demonstrated benefits in alleviating anxiety in the general population and in other caring professions [13–15].

The purpose of this literature review is to describe and analyze the available data on the impact and effectiveness of Reiki therapy in reducing stress in students and healthcare professionals. We hope that the findings will offer insight into Reiki’s potential role in alleviating stress amongst medical students.

## Methods

This literature review was conducted by searching PubMed, Scopus, PsychInfo, Cochrane Library, and gray literature sources for articles investigating Reiki in student and healthcare populations. A total of 363 articles were initially identified.

On PubMed, 97 articles were found using the following search terms: (“Therapeutic Touch” [Mesh]) AND (“Students”) OR (“Health Personnel” [Mesh]). On Scopus, 108 articles were found using the following search terms: (“Therapeutic” AND “Touch” OR “Reiki”) AND (“Students”) OR (“Health” AND “Personnel” OR “Health AND “Professionals” OR “Physicians” OR “Nurses” OR “Physician Assistants”). On APA PsycInfo, 152 articles were found utilizing the following: (“Reiki”) OR (“Therapeutic Touch”) AND (“Students”). From the Cochrane Library, three review articles were retrieved by searching “Reiki” and “Therapeutic Touch”. Additionally, three articles were identified through gray literature sources. Reiki and therapeutic touch were searched on the AAMC MedEd Portal as well as on websites including the National Center for Complementary and Integrative Health, the International Center for Reiki Training, International Association of Reiki Professionals, and The Reiki Alliance.

**Fig. 1:**
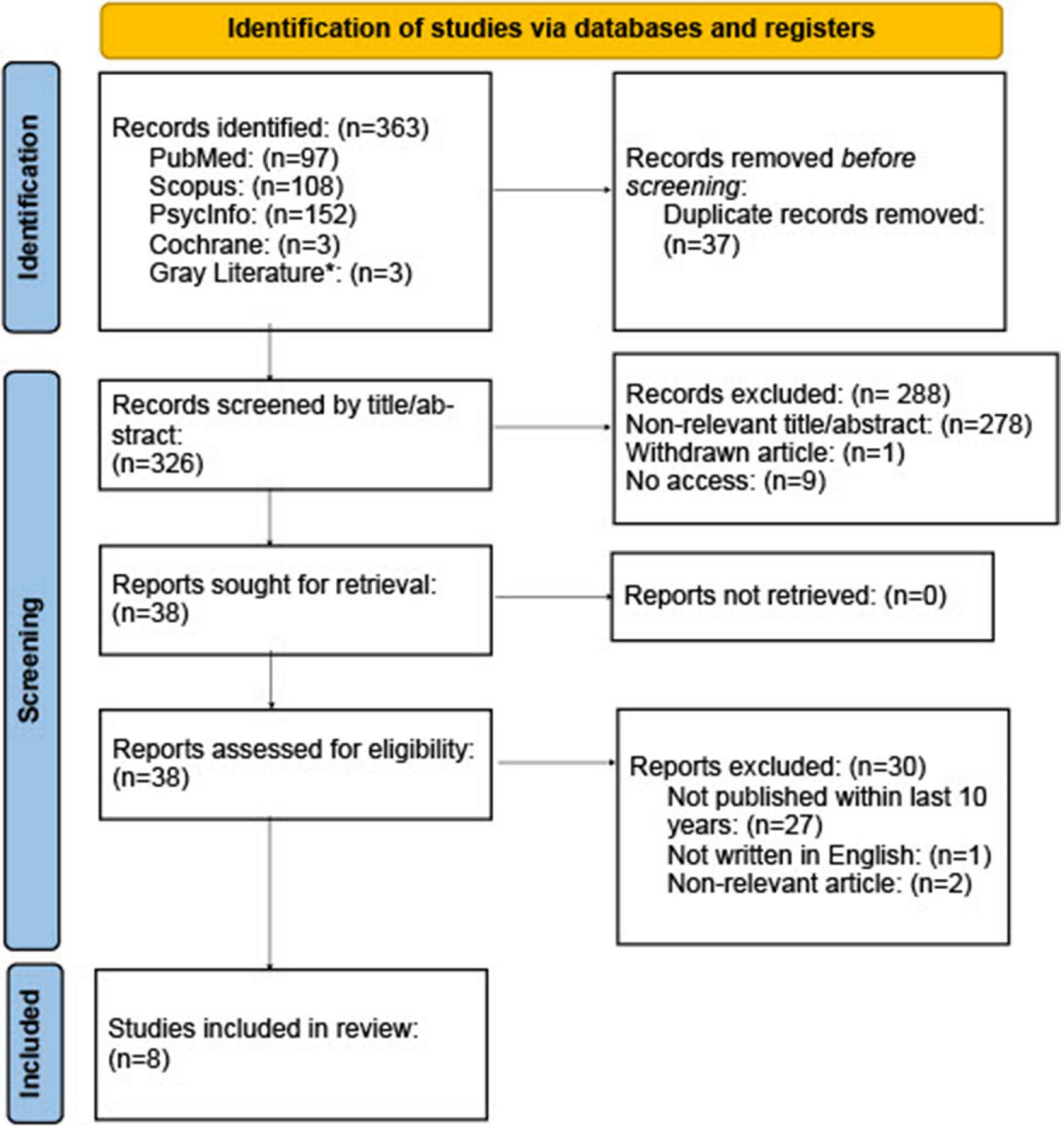
PRISMA Flow Diagram (diagram for articles on Reiki/Therapeutic touch in students and health care populations, which included searches of PubMed, Scopus, PsycInfo, Cochrane and Gray Literature) [16]

After removing 37 duplicates, 326 articles remained and were screened by title and abstract. Inclusion criteria were targeted students and/or healthcare professionals, evaluated therapeutic touch modalities (i.e., Reiki, therapeutic touch) as an intervention, and measured stress or anxiety as an outcome. Articles were excluded if they were published more than 10 years ago or not written in English. Following initial screening, 38 articles were retrieved for full-text review and assessed for eligibility. Of these, 27 were excluded due to publication date, one was excluded for being written in a language other than English, and two were excluded for not meeting the inclusion criteria. Ultimately, eight studies met all criteria and were included in this literature review (Fig. 1).

## Results

Of the eight studies included in this literature review, two were randomized controlled trials [17, 18]; one was a randomized, single-blinded (placebo controlled) cross-sectional experimental study [19]; one was a randomized, single-blinded, repeated measures crossover design [20]; one was a case series [21]; one was an article describing outcomes of nursing students taking a course in complementary and alternative medicine [22]; one employed a correlational research design [23]; and one was a randomized cluster trial [24] (Table 1).

**Table 1:** Summary of characteristics of included articles

First author and year	Country of study	Study design	Sample size; participants	Intervention	Mode of analyzing impact
Bukowski, 2015 (Bukowski, 2015)	The United States of America	Case series	20; undergraduate students	Self administered Reiki	Reiki Baselines Credibility Scale (RBCS), a Reiki Expectancy Scale (RES), Perceived Stress Scale-10 (PSS-10), Global Assessment Questionnaire (GAQ)
Dogru, 2021 (Dogru, Utli & Aykar, 2021)	Turkey	Randomized sham-controlled trial	96; students of nursing and midwifery	Therapeutic touch	Epworth Sleepiness Scale (ESS), Pittsburgh Sleep Quality Index (PSQI), The Perceived Stress Scale (PSS), Visual Analogue Scale for Fatigue (VASF)
Ijaz, 2022 (Ijaz, Rafaq & Shafiq, 2022)	Pakistan	Correlational research design	346; mental health professionals	Self administered Reiki	Teacher Burnout Scale (TBS)
Kramer, 2018 (Kramer, 2018)	The United States of America	Article describing outcomes of students taking a course in complementary and alternative medicine	15; undergraduate nursing students	Self administered energy healing	Journal entries completed by students documenting their experience
Novoa, 2014 (Novoa & Caine, 2014)	The United States of America	Randomized, single blinded (placebo control) cross sectional experimental study	67; mental health professionals	Reiki therapy	Professional Quality of Life: Compassion Satisfaction and Compassion Fatigue/Secondary Trauma subscales, Symptom Questionnaire (SQ), State Hope Scale (SHS): Goal-directed Determination and Planning to Meet Goals
Rosada, 2015 (Rosada et al., 2015)	The United States of America	Randomized, single blinded, repeated measures crossover design	45; mental health clinicians	Reiki therapy	Maslach Burnout Inventory (MBI), Measure Yourself Medical Outcome Profile (MYMOP)
Rosamond, 2023 (Rosamond, 2023)	The United States of America	Randomized cluster trial	150; nurses	Healing touch	Heart rate, blood pressure, respiratory rate, the Visual Analog Scale for Stress (VASS), qualitative exploratory data
Susman, 2024 (Susman, 2024)	The United States of America	Randomized controlled trial	135; undergraduate students	Self compassionate touch	Sussex-Oxford Compassion for the Self Scale Growth mindset: “Kind of Person” Implicit Theory Scale, Positive subscale of the Positive and Negative Affect Schedule (PANAS), Perceived Stress Scale (PSS), DSM-5 Cross-Cutting Measure, Self-Report Behavioral Automaticity Index

All studies involved either students or healthcare personnel. Three studies focused on mental health professionals [19, 20, 23], two on general undergraduate student populations [18, 21], two on nursing or midwifery students [17, 22], and one on practicing nurses [24]. Each study evaluated Reiki or another therapeutic/healing touch modality as the primary intervention.

Four of the eight studies used Reiki specifically [19–21, 23]; of these, two employed self-administered Reiki [21, 23], while the other two used certified Reiki practitioners [19, 20]. The remaining four studies investigated other therapeutic touch modalities, including self-administered energy healing and hands-on techniques developed by Sharon Weiselfish-Giammatteo [22], therapeutic touch [17], healing touch [24], and self-compassionate touch [18].

All eight studies examined the impact of these interventions on stress, though the measurement tools and mechanisms varied. Of the eight studies reviewed, seven were formal research studies, while one [22] was a descriptive article analyzing student journal entries from a course on energy healing. Although not a research study, this article was included to provide a comprehensive overview of the recent work in the field; however, its findings cannot be extrapolated to statistical significance. Amongst the seven research studies, five (71.4%) reported statistically significant reductions in stress.

Rosada et al. [20] examined the impact of Reiki therapy on mental health clinicians using the Maslach Burnout Inventory (MBI) [25], which assesses three components of burnout (emotional exhaustion (EE), depersonalization (DP), and reduced sense of personal accomplishment (PA)). Participants showed a significant reduction in MBI EE scores over time (*b* =  − 1.64, SE = 0.30, *p* < 0.001, 95% CI =  − 2.24 to − 1.03) with Reiki outperforming sham Reiki (*b* =  − 2.03, SE = 0.79, *p* = 0.011, 95% CI =  − 3.58 to − 0.47). MBI DP scores also decreased significantly (estimate =  − 1.50, SE = 0.41, *p* < 0.001, 95% CI =  − 2.31 to − 0.69); however, this effect was observed only amongst unpartnered participants (estimate =  − 63.55, SE = 5.16, *p* < 0.001, 95% CI =  − 73.77 to − 53.34). Sham Reiki reduced DP scores as well, although less effectively (estimate =  − 50.27, SE = 4.38, *p* < 0.001, 95% CI =  − 58.95 to − 41.60). PA scores improved over time (estimate = 0.72, SE = 0.19, *p* < 0.001, 95% CI = 0.34 to 1.10). However, Reiki only showed significant benefit over sham Reiki when administered first (estimate = 25.62, SE = 6.51, *p* < 0.001, 95% CI = 12.57 to 28.67). Rosada et al. also used the Measure Your Medical Outcome Profile version 2 (MYMOP-2) [26] to assess participant-reported symptoms, which showed a significant improvement only amongst single participants (estimate =  − 0.89, SE = 0.38, *p* < 0.001, 95% CI =  − 1.64 to − 0.14).

Bukowski [21] investigated self-Reiki for stress reduction amongst undergraduate students. Self-Reiki is a practice in which individuals apply Reiki to themselves by placing their hands on or near their body. Using the Perceived Stress Scale (PSS), the study found an average reduction of 14.6 points in stress levels from pre to post intervention. Additionally, nearly all participants believed Reiki was a credible and effective stress reduction technique.

Dogru, Utli, and Aykar [17] conducted a randomized controlled trial on therapeutic touch in nursing and midwifery students. Compared to sham therapy and a control group, the intervention produced statistically significant reductions in stress (*p* < 0.001), daytime sleepiness (*p* < 0.0001), and fatigue (*p* < 0.0001), along with improved sleep quality (*p* < 0.001). Ijaz et al. [23] observed a significant reduction in burnout amongst mental health professionals after receiving Reiki (*t* = 1.89, df = 24, *p* < 0.000).

Rosamond et al. [24] evaluated healing touch (HT) in acute care nurses. The randomized controlled trial found a statistically significant reduction in Visual Analog Scale for Stress (VASS) scores post-treatment (− 0.95, *p* = 0.0002) and at follow-up (− 0.73, *p* = 0.0144) compared to a deep breathing control. HT also affected vital signs, with respiratory rate showing near significant reduction post-treatment (1.36, *p* = 0.0568) and a significant reduction at follow-up (− 2.28, *p* = 0.0011).

Kramer [22] described the experiences of nursing students who took a course in complementary and alternative medicine. Students used self-administered energy healing and hands-on touching techniques. Although not a formal research study, journal entries revealed perceived reductions in stress, improved academic performance, greater self-awareness, and increased ability to focus and manage challenges. Students also reported less physical pain and a renewed appreciation for their clinical work and the world around them.

The remaining two articles did not find significant changes following therapeutic touch interventions. Novoa and Caine [19] conducted a randomized, single-blinded, placebo-controlled cross-sectional study on Reiki in mental health professionals. The study did not significantly impact secondary traumatic stress (STS) or its associated symptoms of fear, anxiety, depression, hopelessness, anger, and somatic complaints. Susman et al. [18] studied self-compassionate touch in undergraduates. No significant differences were found between the intervention and active control groups in the intention-to-treat (ITT) analysis (*N* = 135). However, in the per-protocol (PP) analysis (*N* = 45 participants who practiced > 28 times), near-daily practice of SCT led to improvements in self-compassion, stress, and psychopathology.

## Discussion

The majority of the articles analyzed in this review found that Reiki or therapeutic touch significantly reduced stress, burnout, and fatigue in students and healthcare personnel. Two studies did not demonstrate statistically significant improvements, and one article [22], which was not a true research study, could not be evaluated for significance. Nonetheless, the journal reflections in that article indicated that students reported reduced stress after utilizing therapeutic touch.

Stress, burnout, and fatigue are closely interconnected constructs that frequently arise in students and healthcare professionals due to the demanding nature of their work and training [27]. Prolonged exposure to academic or clinical stressors can lead to emotional exhaustion and eventual burnout, which is characterized by marked depersonalization, a diminished sense of personal accomplishment, and persistent fatigue [27]. Fatigue can both result from and perpetuate ongoing stress, contributing to reduced performance, impaired well-being, and emotional disengagement. The high prevalence of these conditions in healthcare environments highlights the need for accessible, low-risk interventions that address both the physiological and psychological dimensions of stress. Reiki and other therapeutic touch modalities are believed to stimulate the body’s relaxation response and promote emotional regulation. These interventions may help interrupt the cycle of chronic stress and reduce symptoms of burnout by providing calming, supportive experiences [12].

While all the included studies focused on energy-based or therapeutic touch interventions, they varied in their specific approaches and methodologies. Some examined traditional Reiki performed by certified practitioners [19, 20], while others assessed self-administered Reiki [21, 23] or related techniques such as therapeutic touch, healing touch, or self-guided energy healing [17, 22, 24]. Though these techniques differ in delivery and style, they share fundamental principles of intentional touch or energy transfer aimed at healing. This thematic overlap justifies their inclusion within a unified review but also introduces variability in outcomes. The mechanisms through which these interventions may operate—such as inducing relaxation, increasing focused attention, providing comforting physical contact, or offering perceived emotional support—likely differ depending on whether the therapy is self-administered or practitioner-administered and based on whether or not physical contact is involved.

Additionally, therapeutic efficacy may be influenced by individual factors such as previous exposure to Reiki, level of receptivity, or personal belief in the therapy. In some studies, sham Reiki and active controls (e.g., deep breathing) led to modest improvements [20, 24], suggesting that therapeutic context and participant expectations may influence outcomes. The fact that sham controls produced statistically significant (though less pronounced) effects compared to true Reiki may offer an important clue into mechanisms at play. One possible explanation is the “enforced relaxation” inherent in both the treatment and the sham conditions. Simply removing oneself from a stress-inducing environment, such as constant study or clinical demands, and engaging in a calm structured session may yield psychological and physiological benefits. This phenomenon may be similar to the stress-reducing effects seen with other interventions such as exercise or meditation. Future research should further examine these variables to disentangle the effects of practitioner presence, physical touch, and placebo responses.

A wide array of tools were utilized across studies to measure stress and burnout, highlighting the diversity of outcome measures and the inherent complexity of researching this topic. Importantly, this is the first review to evaluate the use of Reiki and therapeutic touch specifically in undergraduate students, nursing and midwifery students, and healthcare professionals. While previous systematic reviews have explored Reiki in the context of pain and anxiety in the general population [14, 28], or assessed therapeutic touch for anxiety and depression [29], none has focused on student or healthcare personnel populations. This gap underscores the need for further research, and our findings suggest that Reiki or therapeutic touch may hold promise in reducing stress in these high-risk groups.

### Factors That May Influence Efficacy: Population and Therapeutic Factors

Not all individuals appeared to benefit equally from these interventions. In Rosada et al.’s study [20], Reiki was associated with improvements in all three burnout dimensions (emotional exhaustion, depersonalization, and personal accomplishment), but depersonalization scores improved only amongst unpartnered participants. Similarly, MYMOP-2 scores improved significantly only in single individuals. This suggests that Reiki may offer less additional benefit for individuals who already receive emotional support from a partner. Conversely, unpartnered individuals—who may lack an intrinsic support system—may be more receptive to the benefits of Reiki in reducing burnout.

Additionally, participants with no prior Reiki experience saw greater symptom reductions than those with previous exposure [20], indicating that Reiki may be especially effective in Reiki-naïve individuals. Interestingly, sham Reiki also reduced depersonalization scores, implying that therapeutic factors such as intentional touch, focused attention, or calming environments may contribute to perceived improvements regardless of the specific modality.

### Factors That May Influence Efficacy: Frequency and Timing of Intervention

Therapeutic frequency also appears to influence outcomes. In Susman et al.’s study [18], intention-to-treat analysis showed no difference between self-compassionate touch (SCT) and an active control. However, in the per-protocol analysis, participants who practiced SCT more than 28 times experienced improvements in self-compassion, stress, and psychopathology, suggesting that near-daily practice may be necessary for benefit.

Bukowski [21] found that the most significant reductions in stress occurred during the first 4 weeks of self-Reiki practice, indicating that the intervention’s effects may plateau over time. Rosada et al. [20] observed a potential order effect: Reiki was only significantly more effective than sham Reiki when administered first. This may reflect increased receptivity or novelty associated with receiving Reiki early in the study period.

Given the diversity of populations studied (students, nurses, and mental health professionals) and the variability in intervention types, this review cannot draw definitive conclusions about the comparative efficacy of Reiki versus self-Reiki or other touch modalities across subgroups.

### Strengths and Limitations

A primary strength of this review is that it is the first to focus specifically on Reiki and therapeutic touch in students and healthcare personnel. This offers valuable insight into the potential role of energy-based healing techniques in high-stress populations. These findings may inform future mental health interventions in educational and clinical settings. They may also be relevant to medical students—a group known to experience high rates of anxiety and depression—by introducing a novel and potentially effective wellness intervention. The review underscores the importance of incorporating mental health strategies into medical education.

However, this review also has limitations. The number of eligible studies was small, and the overall quality of evidence varied. One article was not a research study [22], one was observational [21], and one used a correlational design [23], which limits the ability to infer causation. Additionally, the inclusion of sham Reiki—while valuable for comparison—may complicate interpretation, as such interventions may still provide benefit due to the placebo effect or therapeutic setting [19]. Many control conditions also involved calming environments, which could independently reduce stress.

Not all studies included control groups, and some lacked rigorous methodology. Furthermore, although most studies were conducted in the USA, one study was conducted in Turkey and one in Pakistan. These international studies offer valuable insight but may be influenced by cultural perceptions of healing, potentially limiting generalizability to US medical students. Finally, although this review aimed to explore Reiki’s relevance for medical students, no studies to date have examined Reiki in that population directly. Future research is needed to determine the utility, feasibility, and effectiveness of Reiki and other therapeutic touch modalities in medical student populations specifically.

## Conclusion

Reiki has never been formally studied in medical students, despite the high rates of anxiety and depression in this population [1, 2]. This literature review examined existing research in related groups (students and healthcare personnel) to explore Reiki’s potential as a stress-reducing intervention for medical trainees. Most studies reviewed found significant improvements in stress, burnout, or fatigue following Reiki or therapeutic touch interventions. While these findings are promising, they also highlight the paucity of targeted research in medical students. Given the preliminary evidence and the ongoing mental health crisis in medical education, Reiki and other therapeutic touch modalities warrant further investigation in this specific group. Future studies should include larger sample sizes, rigorous control groups, and consistent outcome measures to determine whether Reiki can serve as a viable strategy to support the well-being of medical students.

## References

[1] Quek TT, Tam WW, Tran BX, et al. The global prevalence of anxiety among medical students: a meta-analysis. Int J Environ Res Public Health. 2019;16(15):2735. 10.3390/ijerph16152735. 10.3390/ijerph16152735 PMC6696211 31370266

[2] Puthran R, Zhang MW, Tam WW, Ho RC. Prevalence of depression amongst medical students: a meta-analysis. Med Educ. 2016;50(4):456–68. 10.1111/medu.12962. 26995484 10.1111/medu.12962

[3] GBD results. Institute for Health Metrics and Evaluation. 2021. https://vizhub.healthdata.org/gbd-results/. Accessed 4 Sept 2024.

[4] GBD compare. Institute for Health Metrics and Evaluation. 2021. https://vizhub.healthdata.org/gbd-compare/. Accessed 4 Sept 2024.

[5] The complexities of physician supply and demand: projections from 2021 to 2036. 2024. https://www.aamc.org/media/54681/download. Accessed 4 Sept 2024.

[6] Addressing the physician workforce shortage. AAMC. https://www.aamc.org/advocacy-policy/addressing-physician-workforce-shortage. Accessed 31 Mar 2025.

[7] Yates SW. Physician stress and burnout. Am J Med. 2020;133(2):160–4. 10.1016/j.amjmed.2019.08.034. 31520624 10.1016/j.amjmed.2019.08.034

[8] Babenko O, Daniels LM, Ross S, White J, Oswald A. Medical student well-being and lifelong learning: a motivational perspective. Educ Health (Abingdon). 2019;32(1):25–32. 10.4103/efh.EfH_237_17. 31512589 10.4103/efh.EfH_237_17

[9] Sperling EL, Hulett JM, Sherwin LB, Thompson S, Bettencourt BA. The effect of mindfulness interventions on stress in medical students: a systematic review and meta-analysis. PLoS One. 2023;18(10):e0286387. 10.1371/journal.pone.0286387. 10.1371/journal.pone.0286387 PMC10553303 37796866

[10] Koithan M. Introducing complementary and alternative therapies. J Nurse Pract. 2009;5(1):18–20. 10.1016/j.nurpra.2008.10.012. 20046927 10.1016/j.nurpra.2008.10.012 PMC2754854

[11] Guo X, Long Y, Qin Z, Fan Y. Therapeutic effects of Reiki on interventions for anxiety: a meta-analysis. BMC Palliat Care. 2024;23(1):147. 10.1186/s12904-024-01439-x. 10.1186/s12904-024-01439-x PMC11170819 38872168

[12] Iarp. The science of reiki: understanding the energetic principles. IARP. 2023. https://iarp.org/the-science-of-reiki-understanding-the-energetic-principles/.

[13] Kurebayashi LF, Turrini RN, Souza TP, Takiguchi RS, Kuba G, Nagumo MT. Massage and Reiki used to reduce stress and anxiety: randomized clinical trial. Rev Lat Am Enfermagem. 2016;24:e2834. 10.1590/1518-8345.1614.2834. 10.1590/1518-8345.1614.2834 PMC5172615 27901219

[14] Thrane S, Cohen SM. Effect of Reiki therapy on pain and anxiety in adults: an in-depth literature review of randomized trials with effect size calculations. Pain Manag Nurs. 2014;15(4):897–908. 10.1016/j.pmn.2013.07.008. 24582620 10.1016/j.pmn.2013.07.008 PMC4147026

[15] Özcan Yüce U, Taşcı S. Effect of Reiki on the stress level of caregivers of patients with cancer: qualitative and single-blind randomized controlled trial. Complement Ther Med. 2021;58:102708. 10.1016/j.ctim.2021.102708. 33675935 10.1016/j.ctim.2021.102708

[16] Page MJ, McKenzie JE, Bossuyt PM, Boutron I, Hoffmann TC, Mulrow CD, et al. The PRISMA 2020 statement: an updated guideline for reporting systematic reviews. BMJ. 2021;372:n71. 10.1136/bmj.n71. 33782057 10.1136/bmj.n71 PMC8005924

[17] Vural Doğru B, Hediye Utli, Aykar FŞ. Effect of therapeutic touch on daytime sleepiness, stress and fatigue among students of nursing and midwifery: a randomized sham-controlled trial. Complement Ther Clin Pract. 2021;43:101322. 10.1016/j.ctcp.2021.101322. 10.1016/j.ctcp.2021.101322 33548747

[18] Susman ES, Chen S, Kring AM, Harvey AG. Daily micropractice can augment single-session interventions: a randomized controlled trial of self-compassionate touch and examining their associations with habit formation in US college students. Behav Res Ther. 2024;175:104498. 10.1016/j.brat.2024.104498. 38412573 10.1016/j.brat.2024.104498

[19] Novoa MP, Cain DS. The effects of reiki treatment on mental health professionals who are at risk for secondary traumatic stress. Best Practices in Mental Health: An International Journal. 2014;10(1). 10.31390/gradschool_dissertations.2183.

[20] Rosada RM, Rubik B, Mainguy B, Plummer J, Mehl-Madrona L. Reiki reduces burnout among community mental health clinicians. J Altern Complement Med. 2015;21(8):489–95. 10.1089/acm.2014.0403. 26167739 10.1089/acm.2014.0403

[21] Bukowski EL. The use of self-Reiki for stress reduction and relaxation. J Integr Med. 2015;13(5):336–40. 10.1016/S2095-4964(15)60190-X. 26343105 10.1016/S2095-4964(15)60190-X

[22] Kramer D. Energetic modalities as a self-care technique to reduce stress in nursing students. J Holist Nurs. 2018;36(4):366–73. 10.1177/0898010117745436. 29205082 10.1177/0898010117745436

[23] Ijaz S, Rafaq F, Farah. The effects of Reiki treatment on mental health professionals who are at risk of burnout . Int J Adv Multidisc Res Stud. 2022;2(4):519–22.

[24] Rosamond RL, Giarratano G, Orlando S, et al. Healing touch: a strategy for acute care nurses’ stress reduction. J Holist Nurs. 2023;41(4):347–59. 10.1177/08980101221142193. 10.1177/08980101221142193 36714962

[25] Maslach C, Jackson SE. The measurement of experienced burnout. J Organ Behav. 1981;2(2):99–113. 10.1002/job.4030020205.

[26] Paterson C. Measuring outcomes in primary care: a patient generated measure, MYMOP, compared with the SF-36 health survey. BMJ. 1996;312(7037):1016–20. 10.1136/bmj.312.7037.1016. 8616351 10.1136/bmj.312.7037.1016 PMC2350813

[27] National Academies of Sciences, Engineering, and Medicine; National Academy of Medicine; Committee on Systems Approaches to Improve Patient Care by Supporting Clinician Well-Being. Taking action against clinician burnout: a systems approach to professional well-being. Washington (DC): National Academies Press (US); 2019.

[28] Joyce J, Herbison GP. Reiki for depression and anxiety. Cochrane Database Syst Rev. 2015;2015(4):CD006833. 10.1002/14651858.CD006833.pub2. 10.1002/14651858.CD006833.pub2 PMC11088458 25835541

[29] Robinson J, Biley FC, Dolk H. Therapeutic touch for anxiety disorders. Cochrane Database Syst Rev. 2007;3:CD006240. 10.1002/14651858.CD006240.pub2. 10.1002/14651858.CD006240.pub2 PMC6956493 17636838

